# Antimicrobial and Antifungal Activities of Proline-Based 2,5-Diketopiperazines Occurring in Food and Beverages and Their Synergism with Lactic Acid

**DOI:** 10.1021/acsagscitech.5c00236

**Published:** 2025-08-07

**Authors:** Radek Beneš, Daniel Koval, Ivan Švec, Anna Macůrková, Blanka Vrchotová, Tereza Honzíková, Katsiaryna Kalenchak, Jan Bárta, Veronika Bártová, Jan Bedrníček, František Lorenc, Pavel Smetana, Jan Kyselka

**Affiliations:** † Department of Dairy, Fat and Cosmetics, Faculty of Food and Biochemical Technology, 52735 University of Chemistry and Technology, Technická 3, 166 28 Prague, Czech Republic; ‡ Department of Carbohydrates and Cereals, Faculty of Food and Biochemical Technology, 52735 University of Chemistry and Technology, Technická 3, 166 28 Prague, Czech Republic; § Department of Plant Production, Faculty of Agriculture and Technology, 204731 University of South Bohemia, 370 05 České Budějovice, Czech Republic; ∥ Department of Food Biotechnology and Agricultural Products Quality, Faculty of Agriculture and Technology, 48271 University of South Bohemia, 370 05 České Budějovice, Czech Republic

**Keywords:** 2,5-diketopiperazines, cyclic peptides, antimicrobial activity, antifungal activity, lactic acid, synergism

## Abstract

2,5-Diketopiperazines (2,5-DKPs), naturally occurring in food and beverages, have demonstrated notable antimicrobial properties. However, their synergism with other secondary metabolites in real food matrices remains largely unexplored. In our study, a group of microbially produced 2,5-DKPs, including proline-based dilactams (Pro-DKPs), was synthesized and evaluated for their efficacy against common foodborne pathogens: CCM 4516, CCM 4517, CCM 1961, DBM 4062, CCM 8189, and DMF 0109. We also investigated the impact of the polarity of 2,5-DKPs on their antimicrobial effect. Among the four synthesized 2,5-DKPs, cyclo­(l-Leu-l-Pro) (Pro-DKP-1) inhibited the growth of DMF 0109 by up to 83%, as determined using the poisoned plate method. Further experiments investigated the synergistic effects of Pro-DKPs in combination with lactic acid at food-relevant concentrations. The addition of lactic acid considerably enhanced the antimicrobial activity of all three Pro-DKPs, with inhibitions reaching up to 99% against DMF 0109. Our findings suggest that employing commercial starter cultures capable of producing 2,5-DKPs, including Pro-DKPs, may offer a promising strategy for extending the shelf life of food products and beverages.

## Graphical abstract

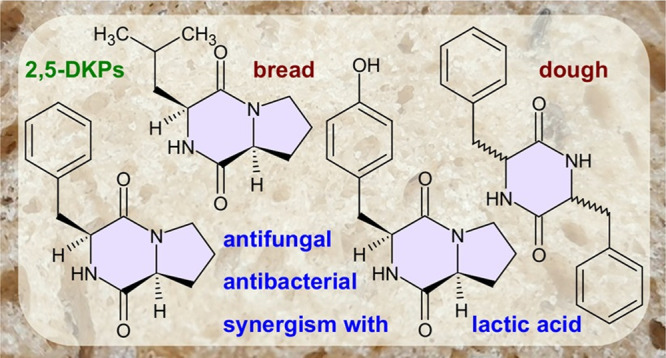

## 1 Introduction

2,5-Diketopiperazines (2,5-DKPs) are the smallest cyclic peptides found in nature, first discovered in the early 20th century. Comprising two amino acid residues attached to a 2,5-DKP ring, they are classified as heterocyclic compounds. Approximately 90% of Gram-negative bacteria are capable of producing these secondary metabolites,^1^ although they have also been isolated from Gram-positive bacteria,^2^ fungi,^3^ higher marine organisms,^4^ and even mammals, including humans.^5^

2,5-DKPs are typically prevalent in processed foods and beverages with high protein content or in fermented products,^6^ as a result of both thermal degradation of amino acids^7^ and microbial biosynthesis, whether by natural or contaminating microflora.^8–10^ Surprisingly, up to 90% of currently known food-derived 2,5-DKPs contain proline (Pro-DKPs) in the form of cyclo­(l-X-l-Pro), as described by Otsuka et al.,^11^ with X representing nonpolar amino acids. It is worth noting that the formation of cyclo­(l-X-l-Pro) and their subsequent epimerization to either cyclo­(l-X-d-Pro) or cyclo­(d-X-l-Pro) can be promoted under mild basic or acidic conditions.^6^ The most important asymmetric Pro-DKPs with hydrophobic amino acid residues (l-Ala, l-Val, l-Ile, l-Leu, l-Phe, l-Tyr, l-Trp) have been detected, for example, in beef,^12^ beer,^13^ bread,^14^ cocoa,^15^ red and white wine,^16^ roasted coffee,^17^ and various types of cheeses.^18^ In 2024, a comprehensive examination of three Japanese *miso*, prepared using different fungal starter cultures ( and ), confirmed the presence of unique dipeptides rich in aspartyl, glutamyl, and lysyl residues, along with 17 previously undescribed 2,5-DKPs.^19^ Pro-DKPs are also consistently found in other processed foods and beverages.

Similar to short-chain linear peptides, 2,5-DKPs are important sensory compounds that contribute to the overall taste of food and beverages. Owing to their hydrophobic nature, they are commonly associated with a bitter, metallic, or salty aftertaste. 2,5-DKPs also exhibit a synergistic effect with other bitter substances, such as theobromine, in cocoa products. Although 2,5-DKPs contribute to the pleasant bitterness of chocolate, André et al. recently reported that they also serve as important markers of cocoa bean processing.^20^ However, apart from cocoa and chocolate, their concentration in food typically does not exceed the taste threshold, as reported by Stark and Hofmann.^15,20^ Specifically, data focusing on Pro-DKPs clearly showed that the bitterness threshold concentration increases with the increasing polarity of the second amino acid residue. For example, cyclo­(l-Phe-l-Pro) exhibited no perceivable bitterness at concentrations below 1020 μmol/L, whereas cyclo­(l-Tyr-l-Pro) had a threshold concentration of 480 μmol/L. Notably, epimerization was found to have no effect on the perception of the bitter taste.^6^

Recently, 2,5-DKPs have gained attention due to their diverse biological activities including antimicrobial properties.^21,22^ The antimicrobial effect of certain 2,5-DKPs can be attributed to their inherent hydrophobic nature. This nonpolar character enables them to disrupt the integrity and function of the outer membrane (in Gram-negative bacteria) or the cytoplasmic membrane (in Gram-positive bacteria and fungi such as and ), ultimately leading to cell death.^23,24^ Moreover, Pro-DKPs such as cyclo­(l-Phe-l-Pro), cyclo­(l-Leu-l-Pro), and cyclo­(l-Tyr-l-Pro) are also part of the quorum sensing system of certain bacteria. Pro-DKPs can, for example, inhibit biofilm formation (e.g., ATCC 29213), thereby making the bacteria more susceptible to other antimicrobial agents.^25–27^ Kumar et al. isolated cyclo­(l-Tyr-l-Pro) and cyclo­(l-Phe-l-Pro) from *Bacillus* sp. and tested them against seed-borne pathogens such as MTCC 183 and MTCC 284.^28^ In these studies, minimum inhibitory concentrations (MICs) of the tested 2,5-DKPs ranged from 41 to 1904 μmol/L. In contrast, studies by Ström et al. and Yan et al. reported significantly higher MICs for 2,5-DKP against similar microorganisms, ranging from 26 to 82 mmol/L.^29,30^

Here, we investigate the antimicrobial effect of three major food-derived Pro-DKPs, namely, cyclo­(l-Phe-l-Pro), cyclo­(l-Leu-l-Pro), and cyclo­(l-Tyr-l-Pro), against selected pathogenic foodborne bacteria and fungi, at concentrations that could realistically occur in food and beverages. For comparison, we also examine the symmetrical, hydrophobic *rac*-cyclo­(Phe–Phe), a dual inhibitor of the serotonin transporter and acetylcholinesterase found in chicken essence.^31^ Unlike previously cited antimicrobial studies, we do not isolate 2,5-DKPs from bacterial cultures but instead synthesize them to exclude potential synergism with other bioactive compounds. Additionally, we assess the antimicrobial activity of four 2,5-DKPs in a real food matrix: bread slices. Given that 2,5-DKPs naturally occur in fermented food, their synergism with lactic acid is also evaluated.

## 2 Materials and Methods

### 2.1 Reagents and Materials

1-Hydroxybenzotriazole hydrate (HOBt), l-phenylalanine, l-phenylalanine methyl ester hydrochloride, l-leucine methyl ester hydrochloride, l-tyrosine methyl ester, 1-ethyl-3-(3-(dimethylamino)­propyl)­carbodiimide (EDC), and *N*-(*tert*-butoxycarbonyl)-l-proline (Boc-l-proline) were purchased from Sigma-Aldrich. All other reagents and solvents were of analytical grade. Cultivation media were purchased from Oxoid Ltd. (Hampshire, UK).

### 2.2 Synthesis of Symmetric *rac*-Cyclo­(Phe–Phe)

The preparation was based on the study of Manchineella et al.^32^ l-Phenylalanine (5.00 g, 30.3 mmol) was dissolved in ethylene glycol (75 mL) and refluxed at 195 °C for 18 h in a 250 mL three-neck round-bottom flask under a nitrogen atmosphere. After the reaction mixture was cooled to room temperature, the resulting white precipitate was filtered, washed twice with ethylene glycol (2 × 50 mL), and recrystallized from ethanol (30 mL) to obtain *rac*-cyclo­(Phe–Phe). Nuclear magnetic resonance (NMR) spectra (^1^H and ^13^C NMR APT) of the compound were recorded using Bruker Avance 600 and Bruker Avance 500 (Bruker, Inc., Billerica, MA, USA).

#### 2.2.1 *rac*-Cyclo­(Phe–Phe)

The *rac*-cyclo­(Phe–Phe) was obtained in 78.5% (11.9 mmol, 3.50 g) yield as white crystals (mp 296–297 °C). *R*_f_ = 0.69 (chloroform:methanol, 5:1, v/v). EI-MS, *m*/*z* (rel. int.) 294 [M]^+·^ (51), 203 [M–CH_2_C_6_H_5_]^+^ (24), 175 [C_10_H_11_N_2_O]^+^ (38), 91 [C_7_H_7_]^+^ (100); ^1^H NMR (500 MHz, (CD_3_)_2_SO): δ 2.23–2.26 (dd, *J*_1_ = 13.6 Hz, *J*_2_ = 6.2 Hz, 2H, Phe-Hβ), 2.57–2.60 (dd, *J*_1_ = 13.6 Hz, *J*_2_ = 4.8 Hz, 2H, Phe-Hβ), 3.97–3.99 (m, 2H, Phe-Hα1, Phe-Hα2), 7.04–7.05 (d, *J* = 7.2 Hz, 4H, Phe-Hδ1, Phe-Hδ2, Phe-Hδ3 and Phe-Hδ4), 7.13–7.14 (d, *J* = 6.5 Hz, 4 H, Phe-Hδ1, Phe-Hδ2, Phe-Hδ3 and Phe-Hδ4), 7.22–7.30 (m, 6H, Phe-Hε1, Phe-Hε2, Phe-Hε3, Phe-Hε4, Phe-Hζ1, Phe-Hζ2), 7.92 (br s, 2H), 8.04 (br s, 2H, NH); ^13^C NMR (126 MHz, (CD_3_)_2_SO): δ 37.70 (Phe-Cβ), 39.36–39.63 (Phe-Cβ), 54.58 (Phe-Cα), 55.39 (Phe-Cα), 126.45–126.57 (Phe-Cζ), 127.93–128.15 (Phe-Cδ), 129.79–130.06 (Phe-Cε,), 135.92 (Phe-Cγ), 166.14 (C=O, C2), 166.81 (C=O, C5).

### 2.3 Synthesis of Asymmetric Proline-Based Cyclo­(l-Phe-l-Pro), Cyclo­(l-Tyr-l-Pro), and Cyclo­(l-Leu-l-Pro)

The preparation of Pro-DKPs was based on the method described by de Costa et al. with slight modifications.^33^ A mixture of Boc-l-proline (5.00 g, 23.0 mmol), l-leucine methyl ester hydrochloride (4.18 g, 23.0 mmol), EDC (6.1 mL, 27.9 mmol), HOBt (3.77 g, 27.9 mmol), and triethylamine (6.5 mL, 46.6 mmol) was dissolved in dichloromethane (10 mL) and stirred at room temperature under a nitrogen atmosphere in a 250 mL round-bottom flask for 24 h. The reaction mixture was then transferred to a separatory funnel by using ethyl acetate (250 mL). The organic phase was washed sequentially with distilled water (200 mL), 5% aqueous citric acid (4 × 60 mL), and 10% aqueous K_2_CO_3_ (2 × 100 mL), and dried over anhydrous sodium sulfate, and the solvent was removed on a vacuum rotary evaporator to yield the intermediate Boc-l-Pro-l-Leu-OMe (6.14 g, 22.04 mmol) as colorless crystals in 78% yield.

The protected linear dipeptide Boc-l-Pro-l-Leu-OMe (6.14 g, 22.04 mmol) was dissolved in trifluoroacetic acid (10 mL, 130.7 mmol) and stirred at room temperature for 1 h. Upon completion, the unreacted trifluoracetic acid was evaporated in vacuo to obtain salt l-Pro-l-Leu-OMe · CF_3_COOH as a colorless oil (6.4 g, quantitative).

The salt l-Pro-l-Leu-OMe · CF_3_COOH (6.4 g, 17.9 mmol) was dissolved in methanol (50 mL), and triethylamine (10 mL, 71.6 mmol, 4 equiv) was added. The reaction mixture was refluxed overnight under a nitrogen atmosphere. After completion of cyclization, solvents were evaporated in vacuo, and the oily residue was dissolved in hot propan-2-ol (20 mL). After cooling to room temperature, cyclo­(l-Leu-l-Pro) began to crystallize spontaneously. The syntheses of cyclo­(l-Phe-l-Pro) and cyclo­(l-Tyr-l-Pro) were done in the same manner.

#### 2.3.1 Cyclo­(l-Leu-l-Pro) (Pro-DKP-1)

The cyclo­(l-Leu-l-Pro) was obtained in a 42.6% (9.8 mmol, 2.06 g) overall yield as white crystals (mp 162–163 °C). *R*_f_ = 0.62 (hexane:diethyl ether:formic acid, 80:20:2, v/v/v). EI-MS, *m*/*z* (rel. int.) 210 [M]^+^^·^ (1), 195 [M–CH_3_]^+^ (3), 167 [M–(CH_3_)_2_CH]^+^ (5), 154 [M–(CH_3_)_2_CH–CH_2_ + H]^+^ (100), 70 [C_4_H_8_N]^+^ (49); ^1^H NMR (500 MHz, CD_3_OD): δ 0.96 (d, *J* = 6.6 Hz, Leu-Hδ2), 1.15 (d, *J* = 6.1 Hz, 3H, Leu-Hδ1), 1.31 (t, *J* = 7.3 Hz, 1H, Leu-Hγ), 1.50–1.53 (m, 2H, Leu-Hβ2), 1.87–1.92 (m, 1H, Pro-Hγ2), 1.93–2.05 (m, 1H, Pro-Hγ1), 2.28–2.32 (m, 2H, Pro-Hβ1), 3.21 (q, *J* = 7.3 Hz, 2H, Pro-Hβ2), 3.92 (t, *J* = 6.1 Hz, 1H, Leu-Hα), 4.26 (t, *J* = 7.9 Hz, 1H, Pro-Hα); ^13^C NMR (126 MHz, CD_3_OD): δ 22.20 (Leu-Cδ2), 23.29 (Leu-Cδ1), 25.76 (Leu-Cγ), 29.07 (Pro-Cβ), 39.40 (Leu-Cβ), 46.43 (Pro-Cδ), 54.63 (Leu-Cα), 60.27 (Pro-Cα), 168.9 (C=O, C5), 172.8 (C=O, C2).

#### 2.3.2 Cyclo­(l-Phe-l-Pro) (Pro-DKP-2)

The cyclo­(l-Phe-l-Pro) was obtained in a 58.9% (13.6 mmol, 3.32 g) overall yield as white crystals (mp 127–128 °C). *R*_f_ = 0.75 (hexane:diethyl ether:formic acid, 80:20:2, v/v/v). EI-MS, *m*/*z* (rel. int.) 244 [M]^+^^·^ (49), 153 [M–C_6_H_5_CH_2_]^+^ (47), 125 [M–C_6_H_5_CH_2_–C=O]^+^ (100), 91 [C_7_H_7_]^+^ (40), 70 [C_4_H_8_N]^+^ (39); ^1^H NMR (500 MHz, CD_3_OD): δ 1.78–2.10 (m, 3H, Pro-Hγ1, Pro-Hγ2, Pro-Hβ2), 3.20 (d, *J* = 7.1 Hz, 2H, Phe-Hβ1, Phe-Hβ2), 3.51–3.55 (m, 1H, Pro-Hδ2), 3.71–3.85 (m, 2H, Pro-Hδ1, Phe-Hβ1), 4.07 (t, *J* = 7.4 Hz, 1H, Pro-Hα), 6.55 (br s, 1H, NH), 7.22 (d, *J* = 7.0 Hz, 2H, Phe-Hδ), 7.25–7.30 (m, 3H, Phe-Hε, Phe-Hζ); ^13^C NMR (125 MHz, CD_3_OD): δ 22.77 (Pro-Cβ), 29.37 (Pro-Cγ), 38.16 (Phe-Cβ), 45.96 (Pro-Cδ), 57.67 (Phe-Cα), 60.07 (Pro-Cα), 128.06 (Phe-Cζ), 129.44–129.64 (Phe-Cδ), 131.03 (Phe-Cε), 137.36 (Phe-Cγ), 166.90 (C=O, C5), 170.91 (C=O, C2).

#### 2.3.3 Cyclo­(l-Tyr-l-Pro) (Pro-DKP-3)

The cyclo­(l-Tyr-l-Pro) was obtained in a 55.2% (12.7 mmol, 3.30 g) overall yield as a viscous, oily yellow liquid. *R*_f_ = 0.47 (hexane:diethyl ether:formic acid, 80:20:2, v/v/v). EI-MS, *m*/*z* (rel. int.) 260 [M]^+^^·^ (8), 154 [M–HOC_6_H_4_CH_2_ + H]^+^ (100), 125 [M–HOC_6_H_5_CH_2_–C=O]^+^ (4), 107 [HOC_7_H_7_]^+^ (47), 70 [C_4_H_8_N]^+^ (20); ^1^H NMR (500 MHz, CD_3_OD): δ δ 1.83–2.12 (m, 3H, Pro-Hγ1, Pro-Hγ2, Pro-Hβ2), 3.22 (d, *J* = 7.3 Hz, 2H, Phe-Hβ1, Phe-Hβ2), 3.54–3.58 (m, 1H, Pro-Hδ2), 3.72–3.87 (m, 2H, Pro-Hδ1, Phe-Hβ1), 4.09 (t, *J* = 7.4 Hz, 1H, Pro-Hα), 6.55 (br s, 1H, NH), 6.72 (d, *J* = 7.6 Hz, 2H, Phe-Hε), 7.06 (d, *J* = 7.7 Hz, 2H, Phe-Hδ); ^3^C NMR (126 MHz, CD_3_OD): δ 22.72 (Pro-Cβ), 29.39 (Pro-Cγ), 37.58 (Phe-Cβ), 45.91 (Pro-Cδ), 57.85 (Phe-Cα), 60.04 (Pro-Cα), 116.19 (Phe-Cε), 127.65 (Phe-Cγ), 132.09 (Phe-Cδ), (Phe-Cζ), 157.67 (Phe-Cζ), 166.95 (C=O, C5), 170.76 (C=O, C2).

### 2.4 Investigation of Selected Strains of Lactic Acid Bacteria (LAB), Yeasts, and Molds for the Production of Cyclo­(l-Tyr-l-Pro), Cyclo­(l-Phe-l-Pro), and Cyclo­(l-Leu-l-Pro)

Sterilized De Man, Rogosa, and Sharpe (MRS) broths (100 mL; 121 °C, 15 min, 0.15 MPa) were inoculated with 1% inoculum (v/v) of the selected LAB: 6P1, 7P2, VT2, and BLIV2, all from the collection of the Department of Dairy, Fat and Cosmetics (UCT Prague, Czech Rep.).^34^ LAB in MRS broths were cultured at 30 °C, aerobically, for 5 days. Uninoculated MRS broth served as the control sample. After the cultivation was completed, 45 mL of individual MRS broths was transferred into test tubes, which were then centrifuged (20 °C, 5000 rcf, 10 min). After centrifugation, the supernatants were extracted with ethyl acetate (3 × 40 mL), organic phases were combined, the solvent was evaporated in vacuo, and the residue was weighed. After weighing, the residue was diluted with ethyl acetate (10 mg of residue/mL) and filtered through disposable PVDF filters (0.2 μm), and the aliquot was transferred into a vial for analysis.

Sterilized malt extract (MEA) broths (100 mL; 115 °C, 10 min, 0.15 MPa) were inoculated with 1% inoculum (v/v) of the selected yeasts and molds: DBM 4062 was from the collection of the Department of Biochemistry and Microbiology (UCT Prague, Czech Rep.), CCM 8189 was obtained from the Czech Collection of Microorganisms (Brno, Czech Rep.), DMF 0109, and var. *boulardii* and DMF 1017 were from the collection of the Department of Dairy, Fat and Cosmetics (UCT Prague, Czech Rep.). Selected yeasts and molds in MEA broths were cultured at 30 °C, aerobically, for 5 days. Uninoculated MEA broth served as a control sample. After the cultivation was completed, 45 mL of individual MEA broths was transferred into test tubes, which were then centrifuged (20 °C, 5000 rcf, 10 min). After centrifugation, the supernatants were extracted with ethyl acetate (3 × 40 mL), organic phases were combined, the solvent was evaporated in vacuo, and the residue was weighed. After weighing, the residue was diluted with ethyl acetate (10 mg of residue/mL) and filtered through disposable PVDF filters (0.2 μm), and the aliquot was transferred into a vial for analysis.

Identification and quantification of target 2,5-DKPs in fermentation broths were performed on an Agilent 8890N gas chromatograph (Agilent Technologies, Santa Clara, CA, USA) coupled with a flame-ionization detector (FID) and an HP-5 column (Agilent Technologies, Santa Clara, CA, USA) of 0.25 mm × 30 m; film thickness of 0.25 μm was used. The conditions of the analysis were as follows: volume of injection, 1 μL; split injection, 1:25 at 300 °C; flow of carrier gas (He), 1 mL/min; temperature program of 80–320 °C with a step of 15 °C/min and a final holding time of 5 min; FID detection at 320 °C; flow of H_2_, 40 mL/min; air flow of 450 mL/min, and makeup gas (N_2_) flow of 45 mL/min. Determinations were performed in triplicate. Quantitative analysis was done by GC/FID using calibration curves for each 2,5-DKP. GC/MS (EI) was recorded using an Agilent 8890 GC system, a 5977C EI MSD (Agilent Technologies, Santa Clara, CA, USA), and a PAL3 Series II Autosampler system under the same conditions used for GC/FID.

### 2.5 Preparation of Wheat Toast Breads Containing Symmetric and Asymmetric 2,5-DKPs

Doughs for wheat toast loaves of bread were prepared according to the recipe summarized in Table 1. First, raw materials, including either cyclo­(l-Leu-l-Pro), cyclo­(l-Phe-l-Pro), cyclo­(l-Tyr-l-Pro), or *rac*-cyclo­(Phe–Phe), were mixed using a Farinograph-TS rheometer (Brabender, Germany). 2,5-DKPs were dosed at the concentration of 10 mmol/kg. Wheat bread without added 2,5-DKPs served as a control sample. The amount of added water was adjusted to achieve the dough consistency of 500 Brabender Units (BU), equal approximately to 1.10 N m. Doughs were mixed for 5 min, weighed on laboratory scales, and placed in a plastic bowl covered by a lid placed into a laboratory incubator FTC 90L equipped with automatic steaming (VELP Scientifica, Usmate Velate, Italy), where doughs rested for 30 min at 32 °C in a modified atmosphere with 80% moisture. Doughs were then split into two pieces with identical weights and placed into stainless baking molds–truncated pyramids of square base 80 mm and height 90 mm (volume ca. 750 mL). Occupied baking molds were then put back into the incubator to proof for another 30 min. In the next step, the mold pair was placed into a laboratory oven (Sadkiewicz’s Baking Industry Research Institute, Bydgoszcz, Poland) preheated to 240 °C. Immediately afterward, the oven was steamed with 50 mL of distilled water. Breads were baked for 20 min, carefully removed from the steel molds, and cooled on filter paper for 2 h at laboratory conditions. Total titratable acidity (TTA) of bread crumb was determined by titration with 0.1 M NaOH solution to the equivalence point, and bread crumb pH values were analyzed using a pH probe. Phenolphthalein was used as a pH indicator.

**Table 1:** Recipe for the Preparation of Wheat Toast Breads Containing Symmetric and Asymmetric 2,5-DKPs

raw material	weight (g)	amount (%)
wheat plain flour T530	300	100
fresh yeasts	12	4
sodium chloride *p.a.*	5,1	1,7
sucrose *p*.*a*.	4,5	1,5
margarine (80%)	3	1,0
water	171	57
synthesized 2,5-DKP	10 mmol/kg	

Breads were then sliced under sterile conditions using a knife treated with 96% ethanol and placed into sterile glass plates. The next steps are described in Section 2.7.

### 2.6 Antimicrobial Activity Assay of Symmetric and Asymmetric 2,5-DKPs

Single strains of Gram-positive bacteria ( CCM 4516) and Gram-negative bacteria ( CCM 4517, CCM 1961), all obtained from the Czech Collection of Microorganisms (Brno, Czech Rep.), were selected to test the growth inhibition activity of the prepared 2,5-DKPs–*rac*-cyclo­(Phe–Phe), cyclo­(l-Phe-l-Pro), cyclo­(l-Tyr-l-Pro), and cyclo­(l-Leu-l-Pro). The bacterial cultures used in the antimicrobial efficacy experiments were prepared freshly in a brain heart infusion (BHI) broth at 37 °C and cultivated for 72 h. The initial density of bacterial strains was approximately 10^7^ CFU/mL. Liquid media were supplemented with various concentrations (10, 5, 2.5, and 1.25 mmol/L) of tested 2,5-DKPs and sterilized (121 °C, 15 min, 0.15 MPa) before the cultivation. To the liquid medium containing *rac*-cyclo­(Phe–Phe) was also added Tween 80 (2%, v/v). A spectrophotometric method, using an automatic cultivator/reader PowerWave XS (BioTek Instruments, Winooski, VT, USA), was used for the quantitative detection of the microbial growth on the microtitration plates (Nuns, Roskilde, Denmark). The tested media enriched with 2,5-DKPs were applied to the wells (200 μL) and inoculated with 1% (v/v) inoculum of the selected microorganism. The optical density (OD) of bacterial cultures in the BHI broth was measured at 650 nm for 24 h. Blank experiments were done with microorganisms in the same manner without the addition of growth-inhibitory compounds. Determinations were performed minimally in triplicate. Standard statistical methods (Student’s *t* test) were used to evaluate the results, and differences were considered significant at *p* ≤ 0.05. The growth inhibition activity of single strains by target 2,5-DKPs was calculated from the following equation:

$$\mathrm{Inhibition}=\left(1-\frac{S_{\mathrm{inhibition}}}{S_{\mathrm{blank}}}\right)\times 100\;[\%]$$ 1

where *S* is the area under the growth curve with and without 2,5-DKPs.

### 2.7 Antifungal Activity Assay and Synergistic Effect of Proline-Based 2,5-DKPs with Lactic Acid

The mold strain CCM 8189 was obtained from the Czech Collection of Microorganisms (Brno, Czech Rep.); DMF 0109 was from the collection of the Department of Dairy, Fat and Cosmetics (UCT Prague, Czech Rep.); and DBM 4062 was from the collection of the Department of Biochemistry and Microbiology (UCT Prague, Czech Rep.). Mold strains were selected to test the growth inhibition activity of the prepared 2,5-DKPs. The molds were grown on MEA agar plates at 25 °C for 72 h and maintained with periodic subculturing at 4 °C. The spore suspension for further inoculation was obtained by washing the particular plate with 5 mL of a physiological saline solution with Tween 80 (0.5%, v/v). The concentration of spores was adjusted to 10^5^ spores/mL by proper dilution. MEA agar was supplemented with various concentrations (10, 5, 2.5, and 1.25 mmol/L) of the 2,5-DKPs and sterilized (115 °C, 10 min, 0.15 MPa) before the cultivation. Tween 80 (2%, v/v) was also added to the MEA agar containing *rac*-cyclo­(Phe–Phe). Production of bread containing 2,5-DKPs (10 mmol/kg) was described in Section 2.5. Antifungal activity was determined by monitoring the radial growth of molds on the agar plates and slices of bread with a caliper. Plates with MEA agar were inoculated by 5 μL of the spore suspension (10 ^5^/mL) in the middle of the dish and incubated at 25 °C for 5 days; pure MEA agar was used as the control. Bread slices were placed in sterile glass Petri dishes and inoculated in the middle with 10 μL of spore suspensions. Determinations were performed in triplicate. Standard statistical methods (Student’s *t* test) were used to evaluate the results, and differences were considered significant at *p* ≤ 0.05. Antifungal activities were calculated from the following equation

$$\mathrm{Inhibition}=\left(1-\frac{S_{\mathrm{inhibition}}}{S_{\mathrm{blank}}}\right)\times 100\;[\%]$$ 2

where *S* is the radial growth area (cm ^2^) of molds on the plates/slices of bread with and without 2,5-DKPs.

Lastly, mold strains CCM 8189 and DMF 0109 were selected to test the synergistic inhibition activity of proline-based 2,5-DKPs with lactic acid. MEA agar supplemented with proline-based 2,5-DKPs (10 mmol/L) and lactic acid (40 mmol/L) was sterilized (115 °C, 10 min, 0.15 MPa) before the cultivation. For comparison, MEA agar with only lactic acid (40 mmol/L) was prepared. Plates were inoculated by 5 μL of the spore suspension (10^5^/mL) in the middle of the dish and incubated at 25 °C for 5 days; pure MEA agar was used as the control. Synergistic antifungal activity was determined by monitoring the radial growth of molds on plates with a caliper. Determinations were performed in triplicate. Standard statistical methods (Student’s *t* test) were used to evaluate the results, and differences were considered significant at *p* ≤ 0.05.

### 2.8 Statistical Analysis

The results of all microbial experiments are presented as the mean ± standard deviation. Statistical analysis of the acquired data was conducted using Statistica 14 software (TIBCO Software Inc., USA). The statistically significant effect of varying 2,5-DKP concentrations and the addition of lactic acid on the antimicrobial properties was evaluated using one-way analysis of variance. All statistical analyses were conducted at a significance level of α = 0.05; therefore, differences with *p* ≤ 0.05 were considered statistically significant.

## 3 Results and Discussion

### 3.1 Synthesis of Cyclo­(l-Tyr-l-Pro), Cyclo­(l-Phe-l-Pro), Cyclo­(l-Leu-l-Pro), and *rac*-Cyclo­(Phe–Phe) and Investigation of Selected LAB, Yeast, and Mold Strains for Their Production

2,5-DKPs previously isolated from both Gram-positive and Gram-negative bacteria have been shown to potentiate the antimicrobial activity of other secondary metabolites, and vice versa.^25–30^ Their presence can ultimately influence and improve the shelf life of food and beverages, leading to the conclusion that further systematic investigation is warranted.

The epimerization of *cis*-configured 2,5-DKPs to cyclo­(d-X-l-Pro) adversely affects the structure-to-antimicrobial activity relationship.^28^ Without stereoselective synthesis, the *cis* or *trans* configuration of 2,5-DKPs bearing aliphatic and aromatic substituents cannot be reliably confirmed. Therefore, we prepared three important food-derived Pro-DKPs, along with one symmetric 2,5-DKP (Figure 1), namely: cyclo­(l-Leu-l-Pro) (Pro-DKP-1), cyclo­(l-Phe-l-Pro) (Pro-DKP-2, known as maculosin-2), cyclo­(l-Tyr-l-Pro) (Pro-DKP-3, known as maculosin-1), and *rac*-cyclo­(Phe–Phe), all of which were commercially unavailable in sufficient quantities. We employed preparations including amide bond formation (i) using Boc-l-proline, l-X-OMe, HOBt, EDC, and Et_3_N, followed by the cleavage of the *N*-protective group (ii) of the linear dipeptide Boc-l-Pro-l-X-OMe. The last cyclization of dipeptide (iii), which was catalyzed by Et_3_N, afforded chiral 2,5-DKPs in a high purity (99%) with final isolation yields of 43.0–58.9% (Figure 1). The stereoselective protocol was originally developed for the synthesis of cyclo­(l-Gly-l-Pro) with a 65% yield in the last step.^33^ In our study, we extended the organic approach to other derivatives, achieving even higher overall yields. The stereoselective protocol can be applied to the gram-scale synthesis of Pro-DKPs bearing bulkier and sterically hindered substituents of both polar and nonpolar amino acids that lack a charged side chain. Preparation of *rac*-cyclo­(Phe–Phe) reported herein was provided by direct coupling of two amino acids (iv) in an excellent overall yield of 78.5%, in strong agreement with previously published results.^32^

**Figure 1:**
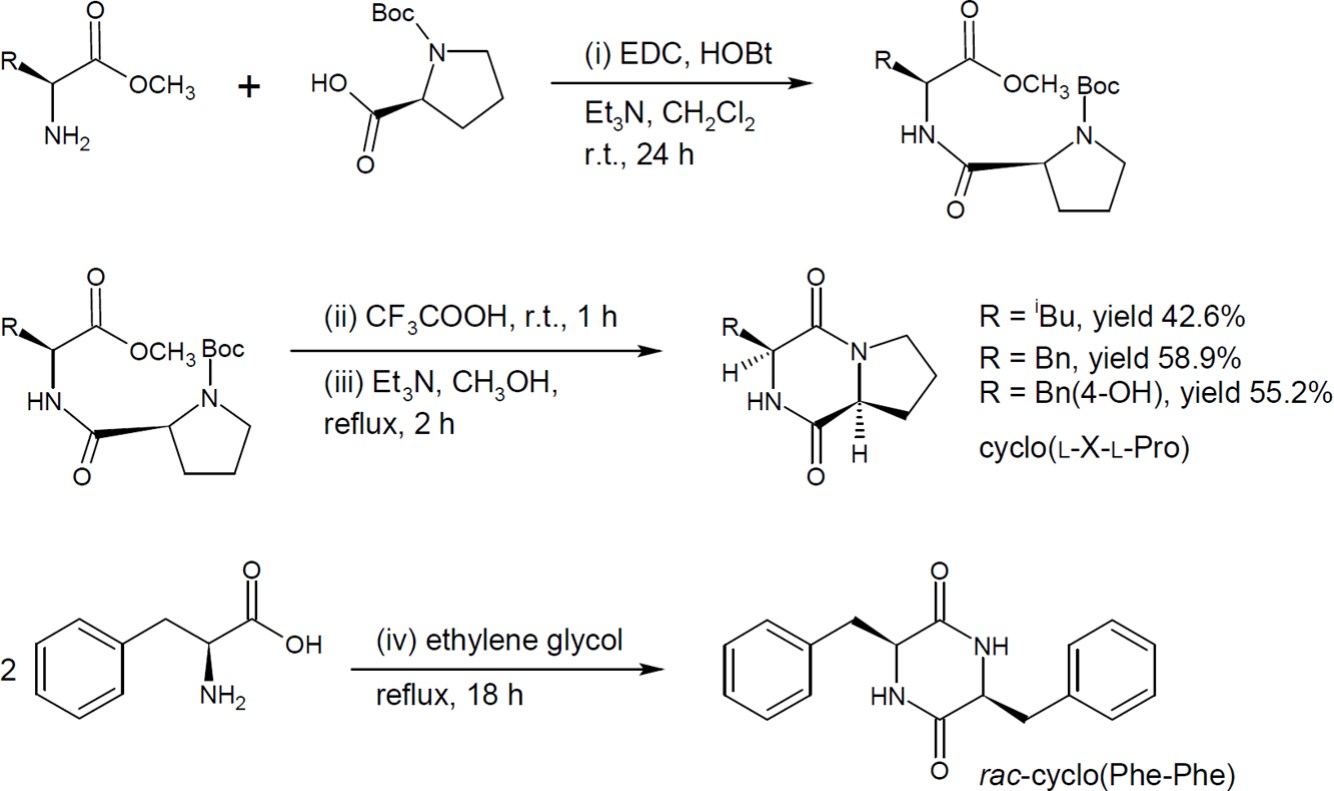
Synthesis of symmetric *rac*-cyclo­(Phe–Phe) and asymmetric proline-based 2,5-DKPs.

Structural elucidation of three *cis*-configured and one *rac*-2,5-DKPs was performed using EI-MS, ^1^H NMR, and ^13^C NMR techniques. The mass spectra of the synthesized analytical standards confirmed the presence of molecular ions with significantly different relative intensities (1–51%), along with fragmentation corresponding to the cleavage of side-chain substituents from the 2,5-DKP ring. In the EI-MS spectrum of cyclo­(l-Leu-l-Pro), we observed a loss of the CH_3_ group (*m*/*z* 195.2), fragmentation of the isopropyl substituent (*m*/*z* 167.2), and a principal side-chain cleavage at the Leu substituent with proton transfer (*m*/*z* 154.2). Fragments at *m*/*z* 125.1 and *m*/*z* 112.1 resulted from partial loss of the six-membered ring, while two diagnostic ions at *m*/*z* 86.2 and *m*/*z* 70.1 corresponded to the immonium ion formed from Leu and Pro, respectively (Figure 2). Additionally, EI-MS spectra of *rac*-cyclo­(Phe–Phe), cyclo­(l-Phe-l-Pro), and cyclo­(l-Tyr-l-Pro) revealed immonium ions corresponding to the phenylalanine (*m*/*z* 120.1) or the tyrosine (*m*/*z* 136.2) moieties. Further elucidation of the six-membered 2,5-DKP ring was carried out by using ^1^H and ^13^C NMR, as shown in Figures 2 and S1 for cyclo­(l-Leu-l-Pro). APT carbon signals of tertiary C (δ 54.63 and 60.27) and quaternary C (δ 168.91 and 172.78) were assigned to the Cα carbon atoms and the carbonyl groups at positions 2 and 5, respectively. These findings were in good agreement with previously published EI-MS, ^1^H NMR, and ^13^C NMR data for 2,5-DKPs.^32,33^

**Figure 2:**
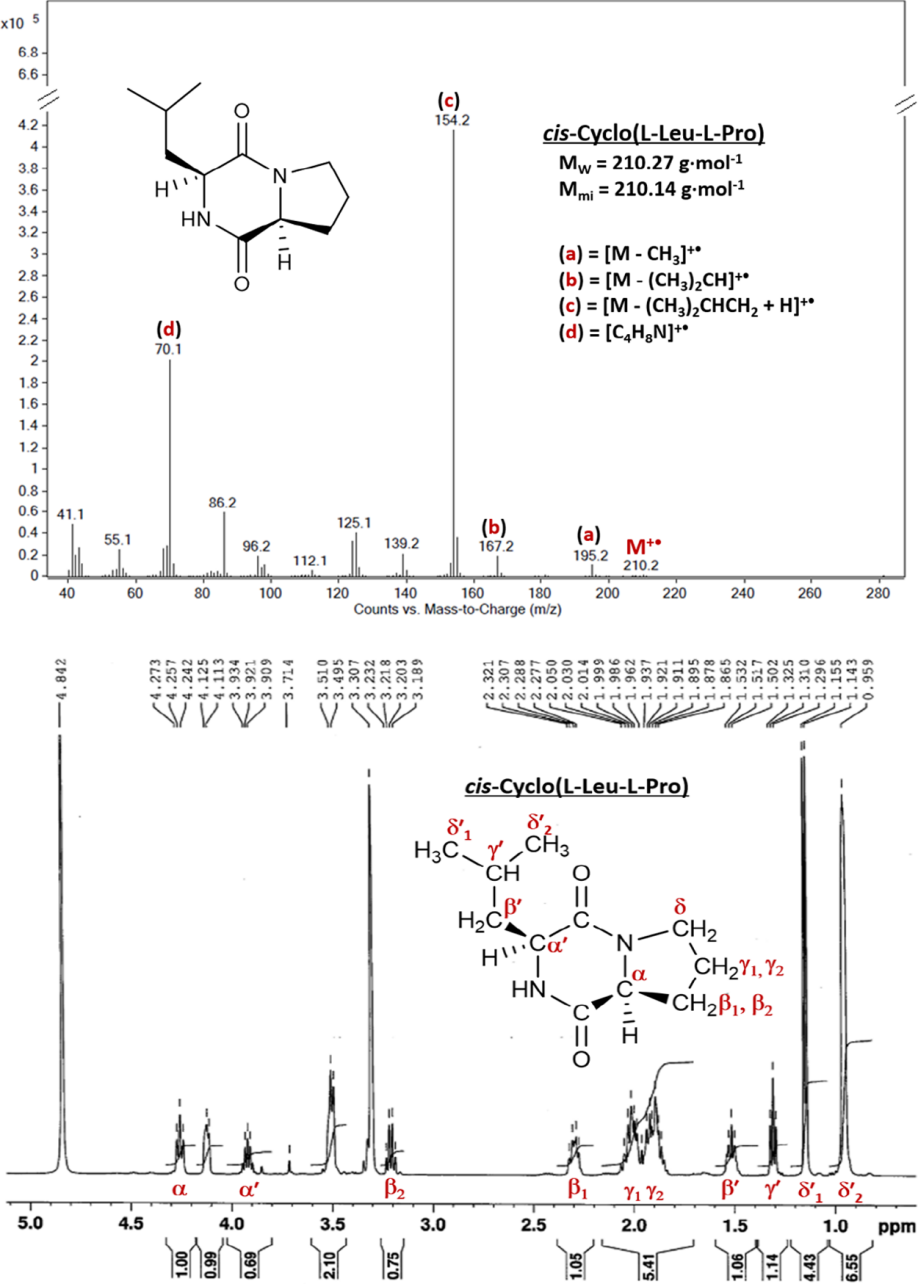
EI-MS (upper image) and ^1^H NMR (lower image) analyses of cyclo­(l-Leu-l-Pro).

We subsequently monitored selected strains of LAB isolated by back-slopping,^34^ as well as technologically relevant yeasts used in dough preparation and mycotoxigenic molds, for their production of Pro-DKP-1, Pro-DKP-2, and Pro-DKP-3 in MRS and MEA broths, which served as model fermentation systems. Notably, production of the tested Pro-DKPs was not observed by pathogenic Gram-positive or Gram-negative bacteria, as discussed in Sections 2.6 and 3.2. To facilitate this work, we developed a robust procedure for isolating the previously synthesized 2,5-DKPs from culture supernatants. The identity of microbially produced 2,5-DKPs was confirmed by comparing their retention characteristics and EI-MS spectra with those of analytical standards. Quantification was performed using GC/FID with calibration curves established for each compound. GC-based separation of 2,5-DKPs was carried out using a 5% phenylmethyl polysiloxane column, yielding the following elution order: cyclo­(l-Leu- l-Pro) > cyclo­(l-Phe- l-Pro) > *rac*-cyclo­(Phe-Phe) > cyclo­(l-Tyr- l-Pro).

Analysis of supernatants revealed lactic, citric, and succinic acids as major constituents, followed by a broad spectrum of secondary metabolites, including the target 2,5-DKPs. We figured out that the highest concentration of cyclo­(l-Tyr-l-Pro) was produced by both *Limosilactobacillus* strains (88.1–102.3 μmol/L) and DMF 0109 (6.9 μmol/L); in both cases, it was the only isolated Pro-DKP metabolite. This represents a novel finding. Additionally, we previously isolated heterofermentative lactobacilli from wholemeal buckwheat sourdough by back-slopping.^34^ The complete spectrum of the investigated 2,5-DKPs was identified only in the supernatant from model fermentation experiments with var. *boulardii* (1.5–7.1 μmol/L), DMF 1017 (2.3–9.5 μmol/L), and 4062 DBM (0.4–1.4 μmol/L), as shown in Table 2. In contrast, only trace concentrations of the tested 2,5-DKPs (0.0–0.4 μmol/L) were detected following fermentation with CCM 8189. Surprisingly, we have refuted any production of dilactams by homofermentative VT2 and BLIV2 strains. This result contradicts published literature data,^35,36^ suggesting that 2,5-DKP biosynthesis was strain-specific.

**Table 2:** Production of Proline-Based 2,5-DKPs by Selected Strains of LAB, Yeasts, and Molds

tested microorganism/concentration of Pro-DKPs (μmol/L)	cyclo (l-Leu-l-Pro)	cyclo (l-Phe-l-Pro)	cyclo (l-Tyr-l-Pro)
6P1	0.0	0.0	102.3 ± 2.3
7P2	0.0	0.0	88.1 ± 2.7
VT2	0.0	0.0	0.0
BLIV2	0.0	0.0	0.0
var. *boulardii*	7.1 ± 1.0	4.5 ± 0.4	1.5 ± 0.4
DMF 1017	9.5 ± 1.0	4.9 ± 0.4	2.3 ± 0.4
CCM 8189	0.0	0.0	0.4 ± 0.0
DMF 0109	0.0	0.0	6.9 ± 1.2
DBM 4062	1.4 ± 0.5	0.8 ± 0.0	0.4 ± 0.0

### 3.2 Determination of Antimicrobial Activities of Synthesized 2,5-DKPs against Pathogenic Gram-Positive and Gram-Negative Bacteria

Next, we discovered the antimicrobial activity of synthesized 2,5-DKPs on the growth inhibition of pathogenic Gram-positive bacteria ( CCM 4516) and Gram-negative bacteria ( CCM 4517, CCM 1961), commonly associated with spoilage in food and feed products, since we refuted the presence of 2,5-DKPs in their supernatants. To evaluate the antimicrobial efficacy of 2,5-DKPs, growth curve data were collected, and inhibitory activities were calculated.

Results presented in Figure 3 show that proline-based 2,5-DKPs had a negligible effect on the growth (inhibition 6.7–19.0%) of CCM 4516 in the concentration range of 1.25–10.0 mmol/L, while *rac*-cyclo­(Phe–Phe) exhibited a dose-dependent effect, with the highest inhibitory activity of 30.8% achieved at 10.0 mmol/L. In fact, our lowest concentration of 1.25 mmol/L approached the content of cyclo­(l-Tyr-l-Pro) found in the supernatant from model fermentation experiments with Gram-positive 6P1 and 7P2 strains. In contrast to the study by Sun et al. (2016), presented derivatives did not reach an MIC as in the case of cyclo­(l-Trp-l-Ser) for ATCC 25923 (11.7 mmol/L).^37^ An explanation can be found in the study reported by Li et al. (2022), who found that cyclo­(l-Phe-l-Pro) at the concentration of 12.3 mmol/L only inhibited the production of virulence factors and the biofilm formation by ATCC 29213, but not microbial growth itself.^25,38^ Thus, the structural motif of synthesized 2,5-DKPs could be recognized by CCM 4516 as a QS inhibitor that influenced cell-to-cell signaling.

**Figure 3:**
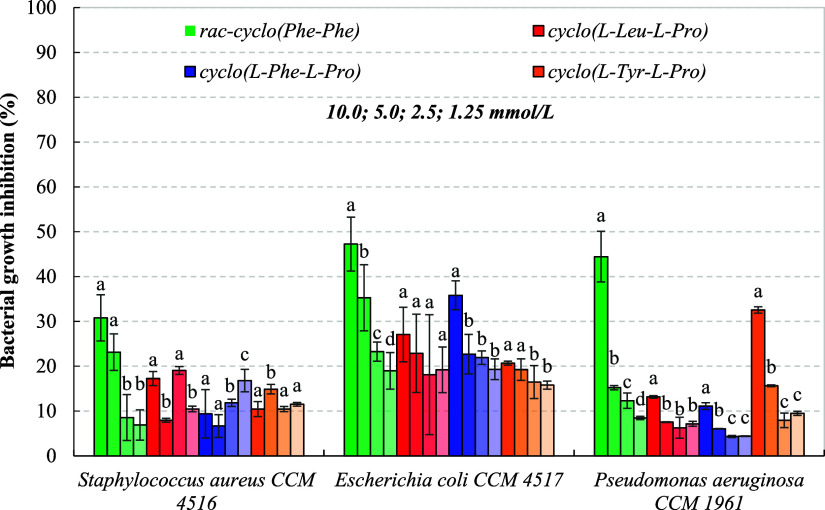
Growth inhibition of selected Gram-positive and Gram-negative bacteria in BHI broth by 2,5-DKPs assayed at 1.25–10.0 mmol/L. Data represent means ± standard deviation of three independent experiments. The darkest shade corresponds to the highest concentration of 2,5-DKPs (10.0 mmol/L), and vice versa (1.25 mmol/L). Bacterial growth inhibition was calculated through eq 1 (see Section 2). Different superscript letters in the columns of particular 2,5-DKP concentrations indicate significant differences (*p* ≤ 0.05) among samples.

Among the selected microorganisms, Gram-negative CCM 4517 was the most sensitive to all assayed (nonpolar) 2,5-DKPs, which most likely disrupted the integrity and function of the outer membrane, leading to a slight protein leakage as determined by the Bradford method at 595 nm. The growth-inhibitory activity of cyclo­(l-Phe-l-Phe) reached 47.3% at 10.0 mmol/L, followed by cyclo­(l-Phe-l-Pro) with an inhibitory activity of 35.8% at the same concentration. It was observed that all 2,5-DKPs decreased the planktonic growth of CCM 4517 in a dose-dependent manner, with an inhibitory activity of at least 15.8% (Figure 3). On the other hand, Gram-negative bacteria CCM 1961 exhibited the highest resistance to the antimicrobial action of the prepared 2,5-DKPs, except for *rac*-cyclo­(Phe–Phe) and cyclo­(l-Tyr-l-Pro) at the highest concentration of 10.0 mmol/L, and growth inhibitions did not exceed 15.6%. Nevertheless, we observed that at a sub-MIC concentration of 10.0 mmol/L, aromatic *rac*-cyclo­(Phe–Phe), cyclo­(l-Phe-l-Pro), and cyclo­(l-Tyr-l-Pro) inhibited the production of QS-regulated virulence factors such as pyocyanin by up to half, as confirmed by UV spectrophotometry. Not surprisingly, a number of current research studies demonstrated the presence of 2,5-DKPs as new signaling molecules isolated from cell-free supernatants of (cyclo­(l-Phe-l-Pro)),^29^ (cyclo­(l-Phe-l-Pro)),^39,40^ and PAO1.^37^ Similar results were achieved in the study of Li et al.,^41^ where cyclo­(l-Phe-l-Pro) and cyclo­(l-Tyr-l-Pro) at 0.4–1.8 mmol/L made no significant difference in bacterial growth between the cyclic dipeptide-treated samples and the blank control; however, the production of typical virulence factors, such as pyocyanin, elastase, proteases, and pyoverdin, as well as biofilm formation, significantly decreased. We postulated that the most common QS autoinducers of Gram-negative bacteria, *N*-acyl homoserine lactones,^37^ and 2,5-DKPs, QS inhibitors commonly found in the *Pseudomonas* genus, could bind to the same regulatory sites and thus affect QS-regulated processes. In general, we discovered that the synthesized 2,5-DKPs, previously identified in real foods and beverages, were present at sub-MIC concentrations.

### 3.3 Determination of Antifungal Properties of 2,5-DKPs against Selected Molds and Their Synergistic Effect with Lactic Acid

Next, we determined whether 2,5-DKPs could serve as templates for creating sustainable biofungicides. CCM 8189, DMF 0109, and DBM 4062 were selected as important spoilage organisms in food systems, where the production of aflatoxins, ochratoxins, and trichothecenes under the optimal growth conditions is of particular human health concern.^29^ Most antifungal peptides target the plasma membrane of molds.^43^ Thus, we aimed to investigate the effect of increasing polarity of synthesized 2,5-DKPs on the mycelium growth inhibition of tested molds on MEA in the presence of 2,5-DKPs (1.25–10.0 mmol/L) for 72, 120, and 168 h at 25 °C. The antifungal activity varied considerably between individual species.

A clear relationship between the polarity of 2,5-DKPs and antifungal activity could only be found in the case of CCM 8189, which represented the most sensitive mold. Our results, shown in Figure 4 (72 h), Figure S2 (120 h), and Figure S3 (168 h), confirmed that the efficacy of synthesized 2,5-DKPs decreased in the following order: *rac*-cyclo­(Phe–Phe) (Log *P* = 3.14) > cyclo­(l-Leu-l-Pro) (Log *P* = 1.50) > cyclo­(l-Phe-l-Pro) (Log *P* = 1.49) > cyclo­(l-Tyr-l-Pro) (Log *P* = 1.09), depending on their increasing polarity. After 72 h of the model experiment, *rac*-cyclo­(Phe–Phe) exhibited the highest mycelium growth inhibition of 47.1–61.5% across the whole tested range, while the most polar cyclo­(l-Tyr-l-Pro) had a considerably lower effect of 25.2–34.3%. The main reason for this was apparently the difference in the transport of individual 2,5-DKPs throughout the plasma membrane of and its ability to biosynthesize a slight amount of cyclo­(l-Tyr-l-Pro) previously shown in Table 2. After 120 and 168 h of cultivation, the adaptation of CCM 8189 to all biofungicides was observed (Figures S2 and S3).

**Figure 4:**
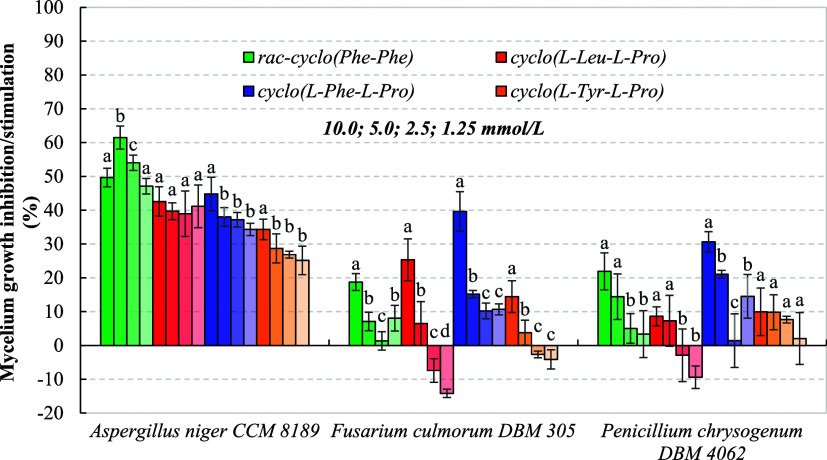
Mycelium growth inhibition of tested molds on MEA with different concentrations (1.25–10.0 mmol/L) of symmetric *rac*-cyclo­(Phe–Phe) and asymmetric proline-based 2,5-DKPs after 72 h at 25 °C. Data represent means ± standard deviation of three independent experiments. The darkest shade corresponds to the highest concentration of 2,5-DKPs (10.0 mmol/L), and vice versa (1.25 mmol/L). The mycelium growth inhibition was calculated through eq 2 (see Section 2). Different superscript letters in the columns of particular 2,5-DKPs concentrations indicate significant differences (*p* ≤ 0.05) among samples.

Interesting groups of microorganisms were Penicillia and Fusaria since they were simultaneously 2,5-DKP-producing species (Table 2) responsible for spoiling various foods, including bread. In general, DMF 0109 and DBM 4062 were less sensitive to the antifungal activity of 2,5-DKPs, with the highest mycelium growth inhibitions of 30.6 and 39.6%, respectively, achieved by cyclo­(l-Phe-l-Pro). In addition, the lowest concentrations of cyclo­(l-Leu-l-Pro) and cyclo­(l-Tyr-l-Pro) even stimulated the growth of Fusaria and Penicillia. Our conclusions corresponded with the ability of DMF 0109 and DBM 4062 to biosynthesize both 2,5-DKPs, as indicated in Section 3.1.

Next, we determined whether some previously undescribed synergism with other microbial secondary metabolites occurs. We investigated the combined effect of cyclo­(l-Leu-l-Pro), cyclo­(l-Phe-l-Pro), and cyclo­(l-Tyr-l-Pro) dosed at 10 mmol/L with food-relevant concentrations of lactic acid (40 mmol/L) against the growth of CCM 8189 representing the most sensitive species and DMF 0109 as a 2,5-DKP-resistant microorganism. The combined effects of proline-based 2,5-DKPs and lactic acid on the mycelium growth of the tested molds after 70, 120, and 168 h on MEA are presented in Figure 5. Surprisingly, CCM 8189 was fully inhibited by the synergistic combination of cyclo­(l-Phe-l-Pro) and lactic acid after 72 h of cultivation; lactic acid on its own exhibited a mycelium growth inhibition of only 6.4%. The combination of cyclo­(l-Leu-l-Pro) and lactic acid also inhibited the growth of CCM 8189 to a great extent, by 67.4% after 72 h and by 62.5% after 120 h. Nevertheless, between the more polar cyclo­(l-Tyr-l-Pro) and lactic acid, no visible synergistic effect was observed after 120 h. It is also worth noting that the combined antimicrobial effects of other proline-based 2,5-DKPs and lactic acid against CCM 8189 diminished over time. On the other hand, DMF 0109 was much more sensitive to the antifungal effect of lactic acid than the CCM 8189, with the mycelium growth inhibitions reaching up to 83.8% after 72 h and 72.7% after 168 h. All Pro-DKPs, combined with lactic acid, further increased the mycelium growth-inhibitory activities above 92.9% against DMF 0109 during the entire 168 h of cultivation period (Figure S4), except for the combination of cyclo­(l-Leu-l-Pro) and lactic acid. Notably, the combined synergistic effect of the most polar cyclo­(l-Tyr-l-Pro) and lactic acid nearly fully inhibited DMF 0109 after 72 h of cultivation, with a whopping 99.0% mycelium growth inhibition.

**Figure 5:**
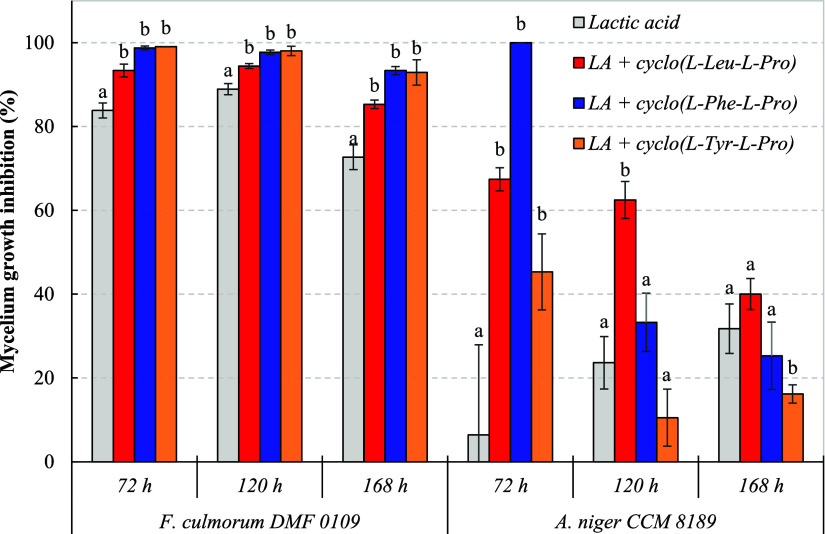
Mycelium growth inhibition of DMF 0109 and CCM 8189 on MEA containing lactic acid (40.0 mmol/L) and proline-based 2,5-DKPs (10.0 mmol/L) after 72, 120, and 168 h at 25 °C. Data represent means ± standard deviation of three independent experiments. The mycelium growth inhibition was calculated through eq 2 (see Section 2). Different superscript letters in the lactic acid column and lactic acid + 2,5-DKP combination column indicate significant differences (*p* ≤ 0.05).

Numerous studies have investigated whether supernatant extracts of , , , , , and other LAB cell cultures containing 2,5-DKPs as principal components could induce cell death or disrupt the cell membrane integrity of foodborne pathogens.^35^ However, the antimicrobial activity of such extracts was primarily attributed to their complex composition, which includes 2,5-DKPs along with other antimicrobial components such as organic acids, which enhance membrane permeability.^35,42^ This assumption was confirmed in the antifungal part of our study and can be regarded as a new contribution to the field. We have also shown that supernatants of selected microorganisms contained assayed 2,5-DKPs; productive Penicillia and Fusaria strains generally exhibited higher resistance to antimicrobial agents and, consequently, higher MICs.

### 3.4 Antifungal Activities of Proline-Based 2,5-DKPs in Wheat Bread Slices

We previously found that the group of Pro-DKPs comprises quite potent antifungal compounds inhibiting the growth of , , and , and thus, they could contribute to the improved shelf life of real food and beverages. Therefore, in addition to solid media, we tested the contribution of proline-based 2,5-DKPs to the overall shelf life of toasted bread as a part of their dough (10 mmol/kg). It is worth noting that cyclo­(l-Phe-l-Pro) and cyclo­(l-Tyr-l-Pro) exceeded the taste threshold concentrations for bitterness of 1.02 mmol/L and 0.480 mmol/L previously described by Stark and Hofmann.^15^ Slices of wheat bread were artificially inoculated with spore suspensions of DMF 0109 and DBM 4062, representing the most prominent industrial contaminants. In addition, aromatic cyclo­(l-Phe-l-Pro), along with cyclo­(l-Leu-l-Pro), belongs to the major 2,5-DKPs found in wheat sourdough and bread, with a concentration in the crust of up to 23 mg/kg. They are formed in situ by fermentation or thermal degradation of proteins during baking.^14^

Bread dough development was evaluated by a Farinograph-TS rheometer (Brabender, Germany) software. Water absorption of bread doughs enriched with LAB and 2,5-DKPs did not significantly change compared with a control bread dough without any additional compounds (data not shown). Dough development time (DDT) and dough stability (S1) of enriched doughs also remained unchanged, and therefore, only one farinograph is shown (Figures 6 and S5). The average DDT reached values of around 3.5 min. Bread crumb analysis of TTA and pH was also conducted. Bread crust was not analyzed because fungi were inoculated in the middle of the bread crumb and their growth is affected by bread crumb acidity. The TTA and pH values of bread without enrichment were 18 and 5.7, respectively.

**Figure 6:**
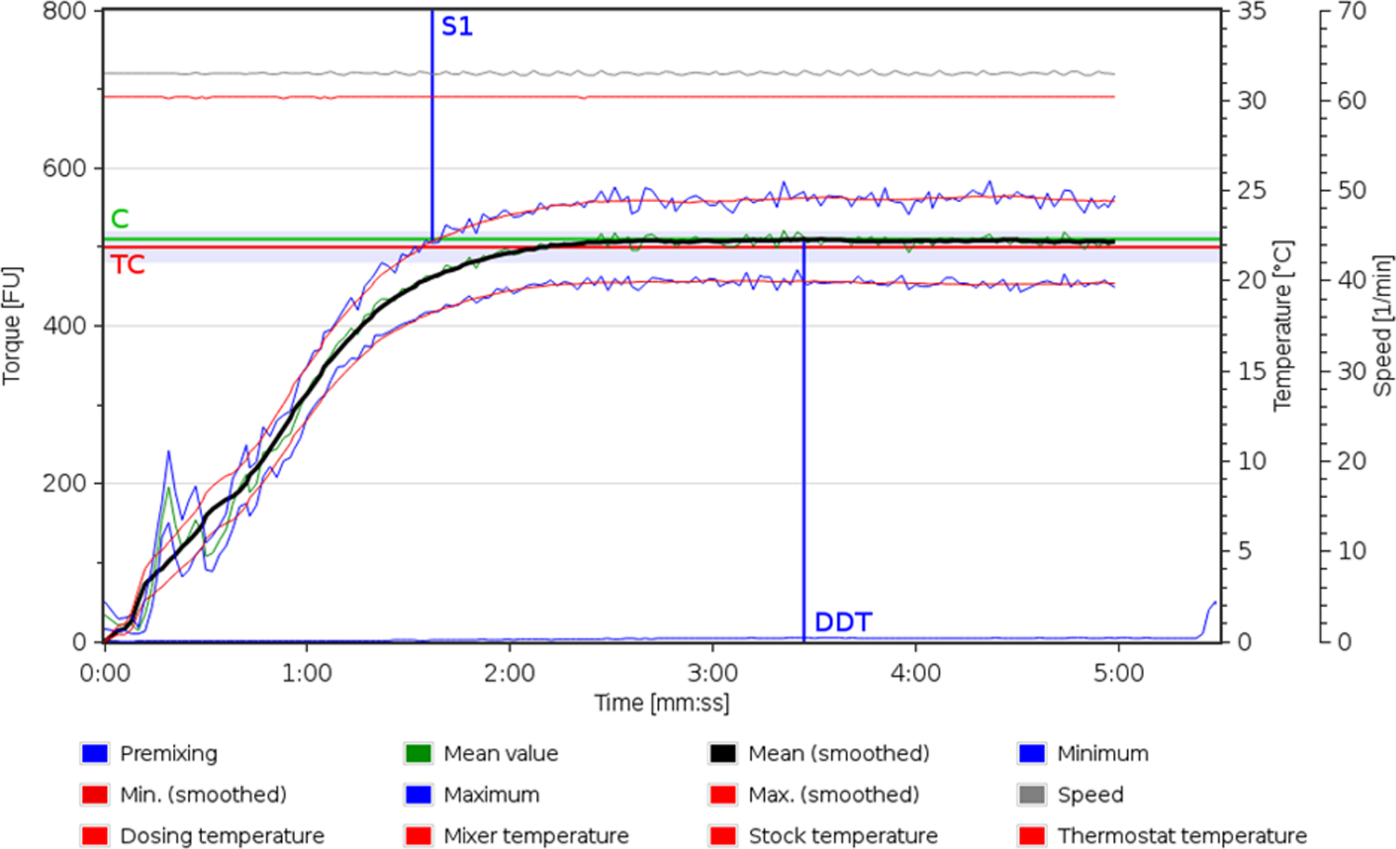
Farinogram of the developed wheat bread dough (DDT 3.5 min).

Generally, proline-based 2,5-DKPs were the most potent after 72 h of mold cultivation. The observed inhibition of mycelia growth was less effective with the extension of the bread storage experiment to 120 and 168 h, respectively (Figure 7). Fungus DMF 0109 was very sensitive to cyclo­(l-Leu-l-Pro) activity, with inhibition reaching up to 83.0% after 72 h, considerably affecting the mold growth, and 53.0% after 168 h, compared to the control (Figure S6). We confirmed the moderate influence of the polarity of the tested compounds on mycelium growth inhibition (Figure 7). The DBM 4062 strain was mostly resistant to the activity of Pro-DKPs. We achieved a promising mycelium growth inhibition of DBM 4062 on bread slices after 72 h using the nonpolar cyclo­(l-Phe-l-Pro), namely, 24.7%. To sum up, our results showed that the prevailing proline-based 2,5-DKPs previously isolated from bread represent biopreservatives with the potential to extend the shelf life of food products. Furthermore, we demonstrated that 2,5-DKPs were resistant to high-temperature treatment during autoclave sterilization (121 °C, 15 min, 0.15 MPa) and bread baking (240 °C, 20 min), as their 10 mmol/kg content was not affected. The effectiveness of the least polar compound, *rac*-cyclo­(Phe–Phe), was generally very low and showed no activity against in a real food matrix.

**Figure 7:**
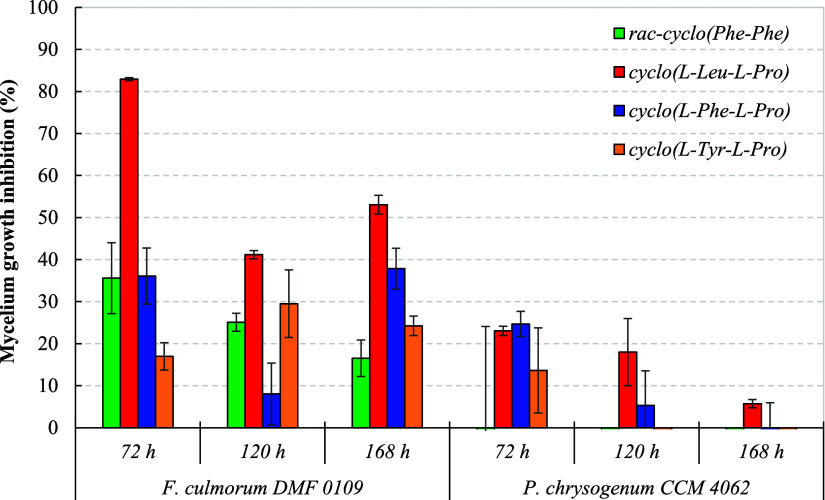
Mycelium growth inhibition of DMF 0109 and DBM 4062 on bread slices containing 10 mmol/kg of the prepared 2,5-DKPs after 72, 120, and 168 h at 25 °C. Data represent means ± standard deviation of three independent experiments. The mycelium growth inhibition was calculated through eq 2 (see Section 2).

In the case of bread as an example of a real food matrix, Pro-DKPs successfully extended its shelf life and inhibited the growth of and . This supports their commercial use for the fortification of bitter foods and beverages, where there is minimal risk that consumers will perceive the resulting organoleptic properties of the final product as undesirable or problematic.

## Supplementary Material



## Notes

^13^C NMR spectra of cyclo­(l-Leu-l-Pro) (Figure S1); mycelium growth inhibition of the tested molds on MEA with different concentrations (1.25–10.0 mmol/L) of symmetric *rac*-cyclo­(Phe–Phe) and asymmetric proline-based 2,5-DKPs after 120 h at 25 °C (Figure S2); mycelium growth inhibition of tested molds on MEA with different concentrations (1.25–10.0 mmol/L) of symmetric *rac*-cyclo­(Phe–Phe) and asymmetric proline-based 2,5-DKPs after 168 h at 25 °C (Figure S3); mycelium growth of DMF 0109 on MEA containing lactic acid (40.0 mmol/L) and proline-based 2,5-DKPs (10.0 mmol/L) after 168 h at 25 °C (Figure S4); farinogram of the developed wheat bread dough (Figure S5); and mycelium growth of DMF 0109 on bread slices containing 10 mmol/kg of the prepared proline-based 2,5-DKPs after 168 h at 25 °C (Figure S6) (PDF)
