# Supplementary material for: Antimicrobial and Antifungal Activities of Proline-Based 2,5-Diketopiperazines Occurring in Food and Beverages and Their Synergism with Lactic Acid
Source: ACS Agric Sci Technol. 2025 Aug 7;5(8):1681–92. doi: 10.1021/acsagscitech.5c00236 (PMC12365876; doi:10.1021/acsagscitech.5c00236)
Supplement: Supplementary file 1 [file as5c00236_si_001.pdf]

TITLE AND AUTHORSHIP

Title:

Antimicrobial and Antifungal Activities of Proline-based 2,5-Diketopiperazines Occurring in Food and Beverages and Their Synergism with Lactic Acid

Authors:

Radek Beneš[1], Daniel Koval[1], Ivan Švec[2], Anna Macůrková[1], Blanka Vrchotová[1], Tereza Honzíková[1], Katsiaryna Kalenchak[1], Jan Bárta[3], Veronika Bártová[3], Jan Bedrníček[4], František Lorenc[4], Pavel Smetana[4], Jan Kyselka[1*]

Affiliations and addresses of the authors:

[1]Department of Dairy, Fat and Cosmetics, Faculty of Food and Biochemical Technology, University of Chemistry and Technology, Technická 3, 166 28 Prague, Czech Republic

[2]Department of Carbohydrates and Cereals, Faculty of Food and Biochemical Technology, University of Chemistry and Technology, Technická 3, 166 28 Prague, Czech Republic

[3]Department of Plant Production, Faculty of Agriculture and Technology, University of South Bohemia, 370 05 České Budějovice, Czech Republic

[4]Department of Food Biotechnology and Agricultural Products Quality, Faculty of Agriculture and Technology, University of South Bohemia, 370 05 České Budějovice, Czech Republic

Email address of the corresponding authors: kyselkaj@vscht.cz

Tel: +420 220 443 266

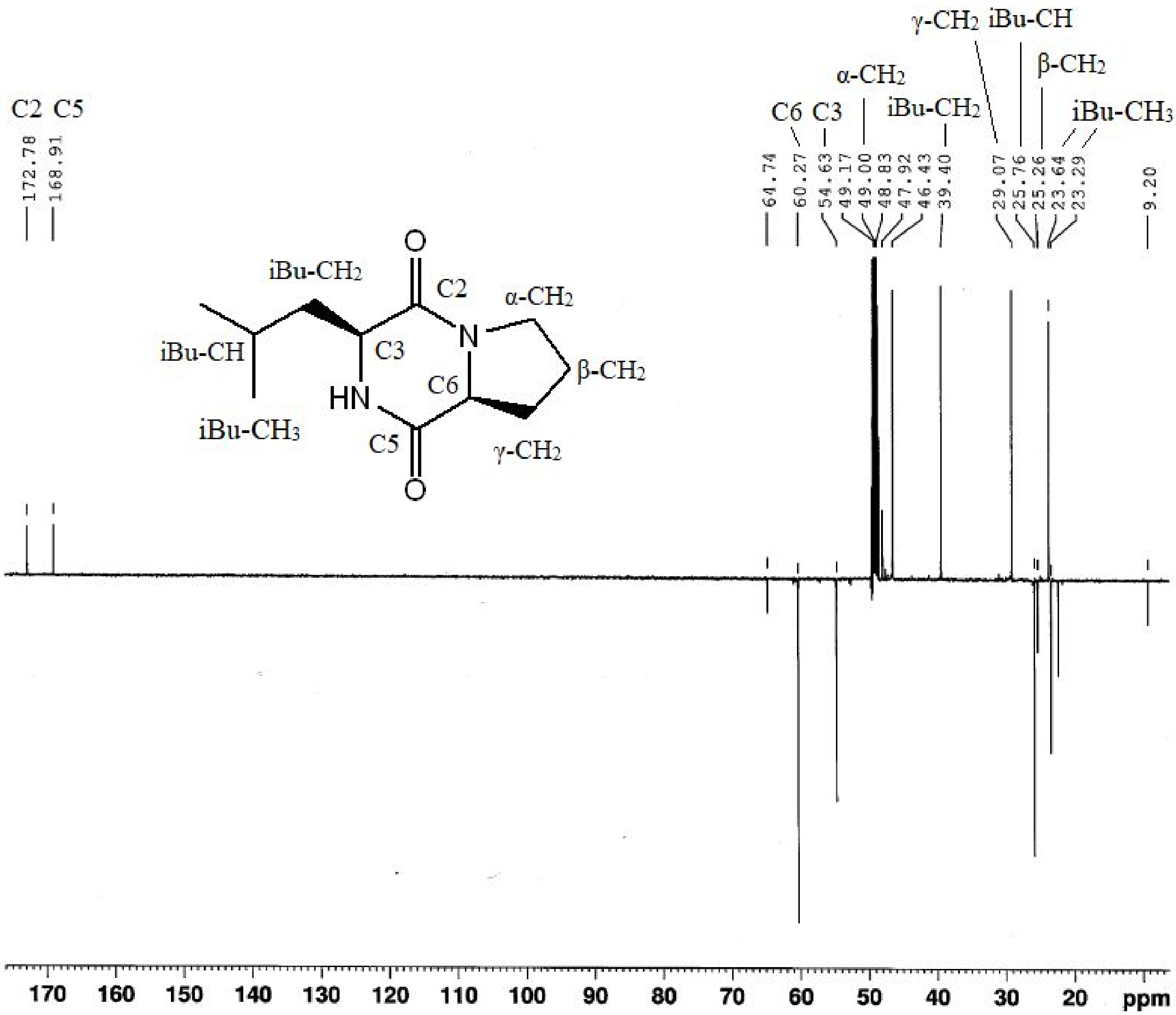

Figure S1. Results of ^{13}C-NMR analysis of cyclo(L-Leu-L-Pro)

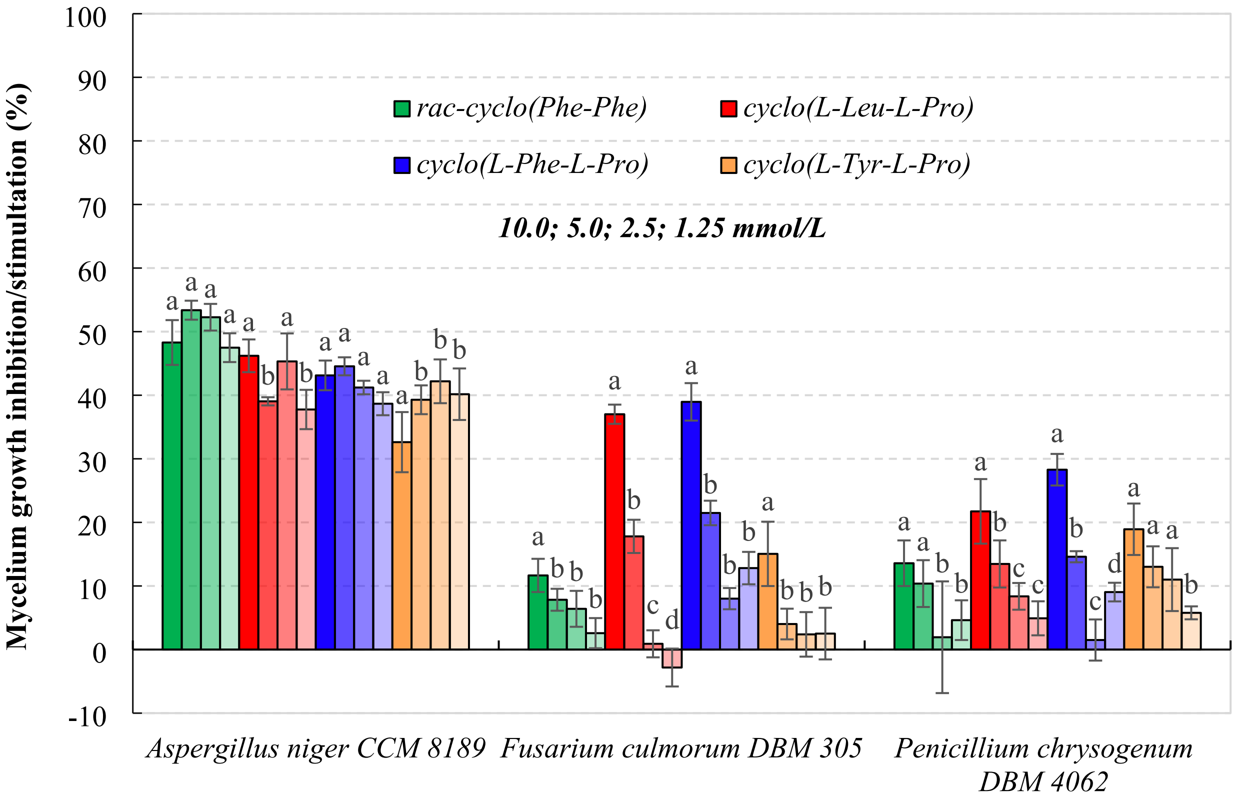

Figure S2. Mycelium growth inhibition of tested molds on MEA with different concentrations (1.25 – 10.0 mmol/L) of symmetric *rac*-cyclo(Phe-Phe) and asymmetric proline-based 2,5-DKPs after 120 hours at 25 °C. Data represent means ± standard deviation of three independent experiments. The darkest shade corresponded to the highest concentration of 2,5-DKPs (10.0 mmol/L) and *vice versa* (1.25 mmol/L). The mycelium growth inhibition was calculated through eq. 2 (see Materials and Methods). Different superscript letters in the columns of particular 2,5-DKPs concentrations indicate significant differences ($p \leq 0.05$) among samples.

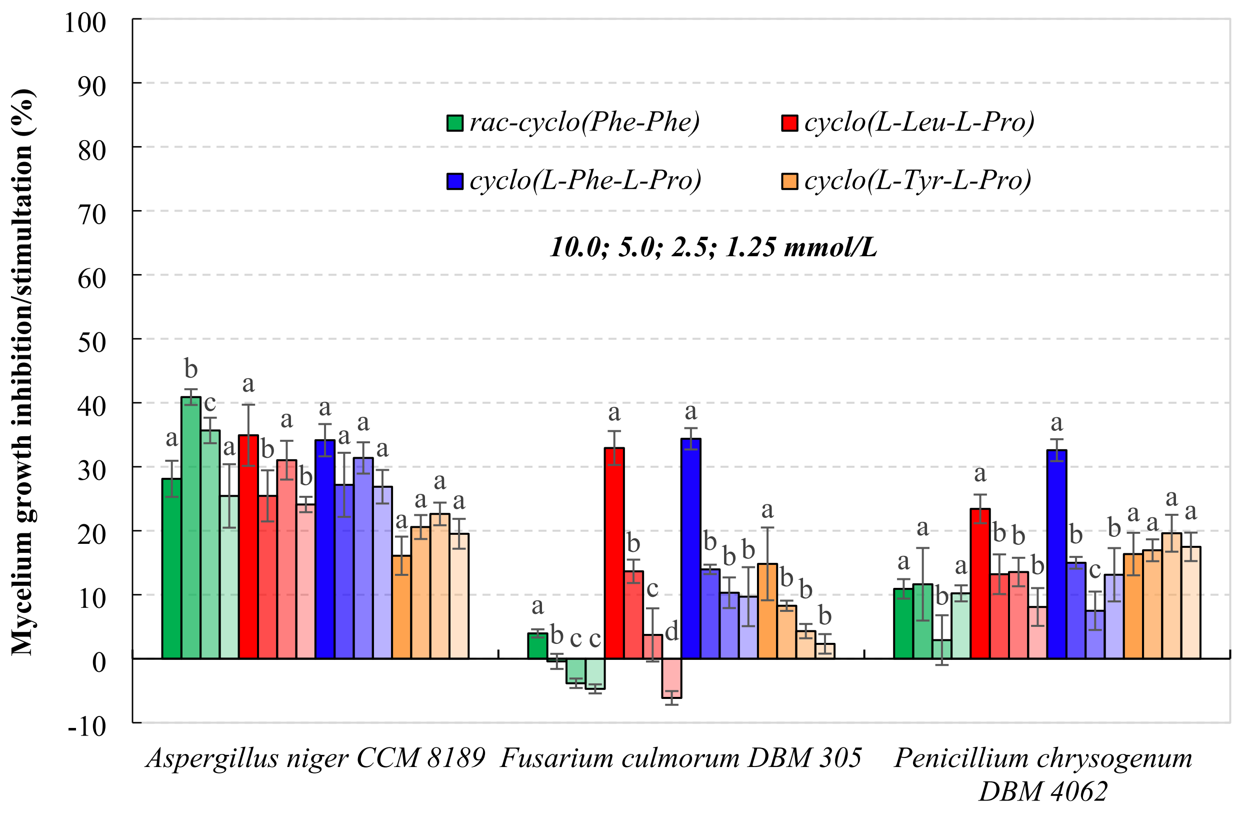

Figure S3. Mycelium growth inhibition of tested molds on MEA with different concentrations (1.25 – 10.0 mmol/L) of symmetric *rac*-cyclo(Phe-Phe) and asymmetric proline-based 2,5-DKPs after 168 hours at 25 °C. Data represent means ± standard deviation of three independent experiments. The darkest shade corresponded to the highest concentration of 2,5-DKPs (10.0 mmol/L) and *vice versa* (1.25 mmol/L). The mycelium growth inhibition was calculated through eq. 2 (see Materials and Methods). Different superscript letters in the columns of particular 2,5-DKPs concentrations indicate significant differences ($p \leq 0.05$) among samples.

cyclo (L-Leu-L-Pro) 10 mmol/L + LA 40 mmol/L

cyclo (L-Phe-L-Pro) 10 mmol/L + LA 40 mmol/L

cyclo (L-Tyr-L-Pro) 10 mmol/L + LA 40 mmol/L

Lactic acid 40 mmol/L

F. culmorum DMF 0109 Control – 168 h

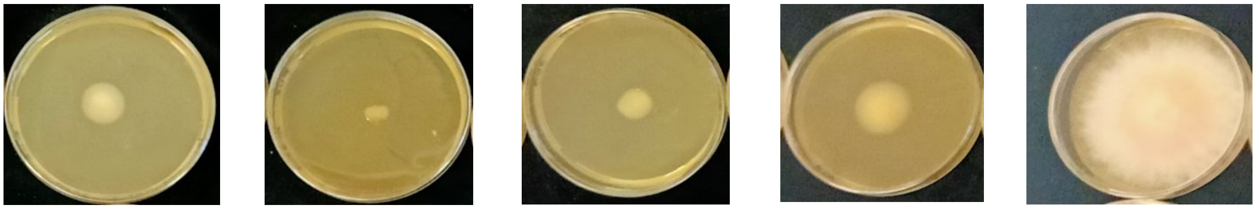

Figure S4. Mycelium growth of *Fusarium culmorum* DMF 0109 on MEA containing lactic acid (40.0 mmol/L) and proline-based 2,5-DKPs (10.0 mmol/L) after 168 hours at 25 °C.

Sample	T530 GoodMills pp kontrolni	ID	B14l4oriv3y4	
Order	Koval~ Benes 2022	Date	27/09/2022 09:23	
User	sveci			
Method	Brabender ICC/ISO			
Evaluation	Brabender_ICC_BIPEA	Speed	63.0	1/min
Mixer	300	Measuring time	05:00	mm:ss
Sample weight	300.0 g			
		Moisture content	14.0	%
Default moisture content	14.0 %	WA (given)	57.0	%
Default consistency	500 FU			
Tags	2022; Koval~Benes;			

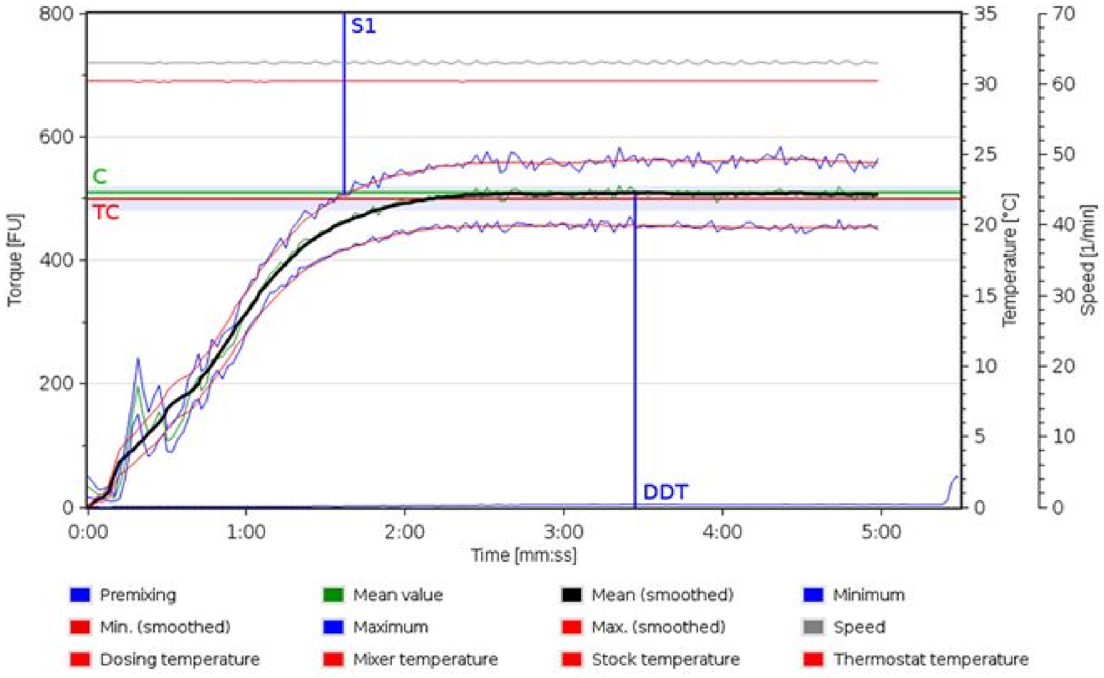

Point	Value	Unit	Description
T	04:59	mm:ss	Measuring time
DT	-/-	°C	Dosing temperature
DDT	03:27	mm:ss	Development time
C	510	FU	Consistency
WZ	57.0	%	Water added
WAC	57.3	%	Water absorption corr. for default consistency
WAM	57.3	%	Water absorption corr. for default moisture content
S	-/-	mm:ss	Stability
DS	-/-	FU	Degree of softening (10 min after beginning)
DS(ICC)	-/-	FU	Degree of softening (ICC / 12 min after max.)
FQN	-/-	mm	Farinograph quality number

Figure S5. Farinogram of developed wheat bread dough (dough development time 3.5 min).

cyclo(L-Leu-L-Pro) 10 mmol/L | cyclo(L-Phe-L-Pro) 10 mmol/L | cyclo(L-Tyr-L-Pro) 10 mmol/L | *F. culmorum* DMF 0109 Control – 168 h

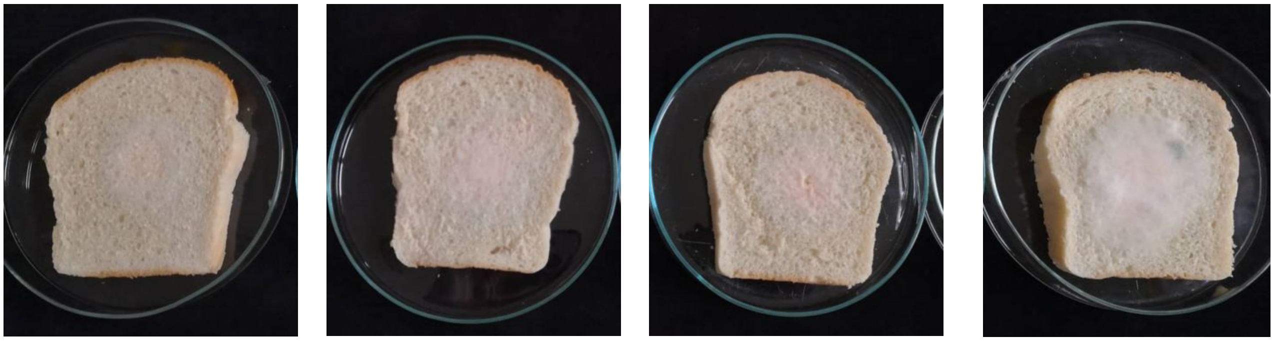

Figure S6. Mycelium growth of *Fusarium culmorum* DMF 0109 on bread slices containing 10 mmol/kg of prepared proline-based 2,5-DKPs after 168 hours at 25 °C.
